# Metachronous Multiple Primary Carcinoma With Acute Promyelocytic Leukemia: 2 Cases Report and Literature Review

**DOI:** 10.3389/fonc.2022.893319

**Published:** 2022-06-08

**Authors:** Cong Wang, Yamei Shen, Yuxia Zhang, Fahui Guo, Qian Li, Huahua Zhang, Xueping Han, Haitao Zhao, Zilong Yang

**Affiliations:** ^1^ Department of Hematology, Wuwei Tumor Hospital, Wuwei, China; ^2^ Wuwei Institute of Hematology, Wuwei, China

**Keywords:** leukemia, promyelocytic, acute, solid tumor, multiple primary carcinomas, diagnosis, prognosis

## Abstract

The co-occurrence of multiple primary cancers with hematological malignancies is uncommon, and acute promyelocytic leukemia (APL) with MPC is even rarer, with only a few cases reported in the literature. Herein, we introduce the diagnosis and treatment of 2 cases of MPC complicated with APL in our hospital and review the relevant literature. Both patients were primary solid tumor patients and were treated with surgery and chemotherapy, and had stable disease (SD). However, more than 1 year after the primary tumor was diagnosed, clinical symptoms were found and APL was diagnosed. Both patients received standard remission-induction therapy, but unfortunately died in the short term due to hemorrhagic complications. In conclusion, treatment of hematological neoplasms, especially acute leukemia combined with multiple primary cancers, is challenging. The prognostic factors and survival analysis of MPC patients with combined APL still need further clinical research and analysis.

## Introduction

Multiple primary cancer (MPC) refers to the occurrence of two or more independent primary malignancies in one or more organs of the same patient, either simultaneously or sequentially. Multiple primary cancers occurring within 6 months of each other are called synchronous carcinoma (SC), while multiple primary cancers occurring more than 6 months apart are called metachronous carcinoma (MC). The diagnostic criteria are: each tumor is histologically malignant; each tumor has its pathological pattern; and there are ≥2 lesions, clearly excluding metastases or recurrence. The combination of multiple primary cancers with hematological tumors is rare, with acute promyelocytic leukemia (APL) combined with multiple primary cancer (MPC) being even more rare, with only a few cases reported in the literature. To discuss the diagnosis, treatment and prognosis of MPC in combination with APL, the data of two patients with multiple primary cancer in combination with APL admitted to our hospital are summarized and analyzed.

## Case Description

**Figure 1:**
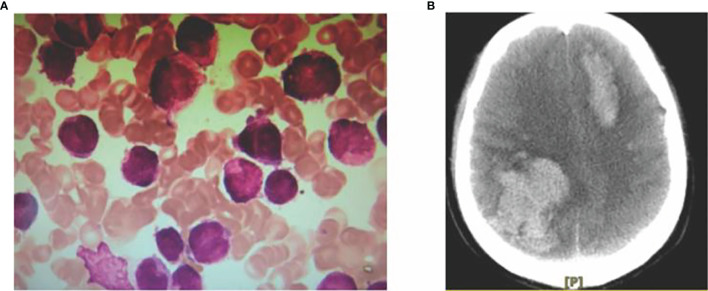
Myeloid morphology **(A)** and CT signs of intracranial hemorrhage in acute promyelocytic leukemia **(B)**.

Case 1 Male, 51 years old, presented to Gansu Cancer Hospital in December 2017 with a clear diagnosis of lung adenocarcinoma due to cough and shortness of breath, underwent right upper lobe lung resection and was given pemetrexed + cisplatin chemotherapy for 6 cycles after surgery, followed by continuous oral gefitinib treatment until this admission. The patient was reviewed several times during this period and the clinical outcome was evaluated as SD. In mid-June 2019 thepatient had frequent gingival bleeding and blood blisters in the buccal mucosa on both sides of the mouth and visited our department on 20 June 2019. Routine blood tests were performed: white blood cells 26.98×109/L, neutrophil count 1.66×109/L, red blood cells 4.84 1012/L, hemoglobin 148 g/L, and platelets 10 x 109/L. Bone marrow aspiration smear: the bone marrow proliferation is obviously active; the granulocyte lineage is abnormally proliferated, 87.5% of the nucleated cells, of which 76.5% are early granulocytes with increased granules, the cells are of different sizes, the nuclei are of various shapes, the nuclei are twisted and folded, small and dense anilinophilic blue granules are seen in the pulp, Auer vesicles in the shape of firewood bundles are easily seen, meganuclei are occasionally seen, platelets are single and rare; blood picture: leukocytes are increased, the early The diagnosis is acute promyelocytic leukemia (APL). Karyotype: 46,XY,t(15;17)(q24;q21),add(18)(q23) [10]. Quantitative PML-RARα fusion gene test: positive, the copy number of PML-RARα fusion gene: 65132 copies. The diagnosis was “acute promyelocytic leukemia (APL)” (**Figure 1**). Treatment regimens for patient was developed according to the 2019 European LeukaemiaNet (ELN)guidelines (1). Retinoic acid 20 mg orally 2 times/day was given from 22 June 2019, and 5 tablets (1.35 g) of compound Huang Dai were added 3 times/day orally from 25 June to induce remission, while intermittent transfusions of plasma, cold precipitation, fibrinogen, platelet supplementation coagulation factors and platelets, and anti-infective, nutritional support, hydration and alkalization, and correction of electrolyte disturbances. On 26 June 2019, the patient developed nausea and vomiting, with 300 ml of stomach contents, followed by confusion and unconsciousness. Emergency cranial CT showed that the right frontoparietal and left frontal lobes had a cerebral hemorrhage, resulting in a local left shift of the midline structures and a small amount of hemorrhage in the subarachnoid space (**Figure 1**). The patient did not recover consciousness and was in a coma. The patient’s family gave up treatment and discharged him from the hospital.

**Figure 2:**
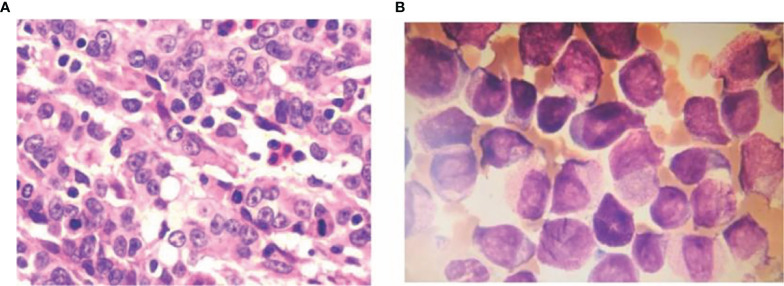
Ulcerated hypofractionated adenocarcinoma of the lesser curvature of the gastric body HE staining 10×40 **(A)** Examination (170259) shows: ulcerated hypofractionated adenocarcinoma of the lesser curvature of the gastric body, Lauren’s staging: diffuse; tumor size 4.5×3 cm. Cancerous tissue infiltrated the plasma layer to the extra-plasma fatty tissue, nerve invasion; cancerous thrombus formation in the lymphatic vessels, no clear cancerous thrombus in the blood vessels; no cancerous tissue was seen in the upper and lower cut edges of the specimen and another sent for examination. No cancerous tissue was seen in the cut margins, the greater omentum (-), regional lymph nodes (0/44) were seen to be metastatic, including (group 1) lymph nodes (0/7), (group 2) lymph nodes (0/4), (group 3) lymph nodes (0/3), (group 4) lymph nodes (0/4), (group 5) lymph nodes (0/0), (group 6) lymph nodes (0/3), (group 7) lymph nodes (0/5), (group 8a) lymph nodes (0/5), (group 9) lymph nodes (0/3), (group 11) lymph nodes (0/0), (group 12) lymph nodes (0/0); immunohistochemistry: P53 (40% positive), P-gp(-), GSTπ (++), TopoII (++), Ki-67 (80% positive), TS (-, C-erbB-2 (-). and bone marrow smear **(B)**.

Case 2, male, 52 years old, the patient visited our hospital in May 2019 with gastric discomfort and was diagnosed with “gastric cancer” by electronic gastroscopy. The postoperative pathological examination (170259) showed: ulcerated hypofractionated adenocarcinoma of the gastric body, Lauren’s staging: diffuse; tumor size 4.5×3 cm; cancerous tissue infiltrated the plasma layer to the extra-plasma fatty tissue, nerve invasion; cancerous thrombus formation in the lymphatic vessels, no clear cancerous thrombus in the vessels. Definite cancer thrombus, no cancerous tissue was observed in the upper and lower cut margins of the specimen and in the other cut margin sent for examination, large omentum (-), cancer metastases were noticed in the regional lymph nodes (0/44), of which (group 1) lymph nodes (0/7), (group 2) lymph nodes (0/4), (group 3) lymph nodes (0/3), (group 4) lymph nodes (0/4), (group 5) lymph nodes (0/0), (group 6) lymph nodes (0/3), (group 7) lymph nodes (0/5), (group 8a) lymph nodes (0/5), (group 9) lymph nodes (0/3), (group 11) lymph nodes (0/0), (group 12) lymph nodes (0/0); immunohistochemistry: P53 (40% positive), P-GP (-), GSTπ (++), TopoII (++), Ki-67 (80% positive), TS (-, C erbB-2 (-) (**Figure 2**). The patient was treated with SOX regimen chemotherapy (oxaliplatin 200mg IV d1, tegafur 60mg oral bid d1-14) for 3 cycles and XELOX regimen (oxaliplatin 200mg IV d1, capecitabine 1.5g oral bid d1-14) for 3 cycles. The patient’s blood count showed 13.34×109/L white blood cells, 3.84×109/L neutrophils, 3.87×1012/L red blood cells, 121 g/L hemoglobin and 34×109/L platelets. bone marrow aspiration results suggested acute promyelocytic leukemia (APL). The flow results were consistent with an acute myeloid leukemia immunophenotype with a high probability of APL. The fusion gene was positive for PML-RARαS subtype (bcr-3) (+) with positive WT1 expression. The diagnosis of “acute promyelocytic leukemia (APL)” was confirmed and the patient was given vincristine 20 mg orally twice/day from 4 December 2020 and augmented with cytarabine 100 mg IV once/day from 7 December to induce remission. The above treatment regimens were determined according to the European LeukemiaNet (ELN) 2019 (1). At 20:30 on 8 December 2020, the patient vomited about 30 ml blood and did not respond to calls. The patient was checked for bilateral pupils about 2 mm, blunted reflex to light and cyanotic petechiae in the left eye sockets. Resuscitation treatment such as hemostasis and dehydration were given, the patient vomited blood again in an amount of about 100 ml. The patient was comatose, sigh-like breathing, bilateral pupils of 2 mm, blunted reflex to light, heart rate of 65 beats per heart rate of 65 beats/min, blood pressure of 95/50mmHg, oxygen saturation between 65% and 88%. After explaining his condition to his family, the patient was discharged after his family refused further resuscitation and the patient died the night after discharge. Summary of 2 cases of MPC combined with APL see **Table 1**.

**Table 1:** Summary of MPC in 2 cases with combined APL.

Case		1	2
Solid tumor	Type	Adenocarcinoma of the lung	Hypofractionated adenocarcinoma of the stomach
	Treatment modality	Surgery + chemotherapy	Surgery + chemotherapy
	Chemotherapy drug	Pemetrexed + cisplatin, gefitinib	Oxaliplatin + Tegeo, Oxaliplatin + Capecitabine
Diagnosis of APL		18 months after treatment	17 months after treatment
Diagnostic basis		MICM	MICM
Cytogenetic characteristics		t (15;17) (q24;q21),add (18) (q23)	t (15;17) (q24;q21)
Molecular characteristics		PML-RARα fusion gene: positive	PML-RARαS subtype (bcr-3) (+) with positive WT1 expression.
Therapeutic regimen		Retinoic acid, compound Huang Dai	Retinoic acid, cytarabine
APL survival time		15days	15days

## Discussion

Multiple primary carcinomas mainly occur in organs with similar tissue types, such as the upper respiratory tract, upper gastrointestinal tract and genitourinary system. The incidence of multiple primary carcinomas in combination with solid tumors in the hematologic system is rare, with only 0.1% reported by Xu Hao et al. (2) in China and 0.5% reported by Cuit et al. (3) abroad. Moertel et al. (4) reported only 9 cases of acute myeloid leukemia (AML) among 194 patients with multiple primary cancers with haematological malignancies, all of which were non-APL subtypes. Xie Xiaoyan et al. (5) reported 6 cases of solid tumors combined with acute leukemia, including 2 cases with M2, 1 case each with M5, M3 and M4, and 1 case with AML that could not be classified (**Table 2**).

**Table 2:** Clinical profile of patients with MPC combined with APL reported in the literature, 2000-2020.

Author	Year	NO.	Age	Gender	Solid tumours			APLTreatment		Prognosis
					Part	Pathology	Staging	Treatment		
Mi Rui Hua et al. (6)	2020	2	58	Female	Breast	Invasive ductal carcinoma	Stage II	Surgery + chemotherapy	Tretinoin 、As2O3、IA、HA、MA、DA	Stable follow-up visits
			52	Female	Esophagus	Squamous cell carcinoma	Stage I	Surgery	Tretinoin、As2O3、IA、HA、ID-Ara-cx2	Stable follow-up visits
ShaLiu, et al. (7)	2019	1	56	Male	Esophagus	Squamous cell carcinoma	Stage IIIB	Surgery + chemotherapy + radiotherapy	Tretinoin、As2O3	Died
Wu Zhijun et al. (8)	2009	1	54	Female	Ovarian	Mucinous cystic adenocarcinoma	Stage III C	Surgery + chemotherapy	Tretinoin	Died
Li Weibin et al. (9)	2002	1	55	Male	Gastric	–	–	Surgery + chemotherapy	–	Died

HA high trichostatin (HHT) in combination with cytarabine (Ara-C); ID-Ara-C is medium-dose cytarabine; MA is mitoxantrone in combination with cytarabine; As2O3 is arsenic trioxide; IA is idarubicin (IDA) in combination with cytarabine; DA is erythromycin (DNR) in combination with cytarabine.

It is well documented that the pathogenesis of MPC is multifaceted with genetic abnormalities, regional theories of carcinogenesis, infections, therapeutic factors, tumor immunity and *in vivo* hormones. It is well established that radiation therapy can lead to secondary tumourigenesis, especially exposure to brain, thyroid, breast, skin, bone and soft tissue. Systemic anti-tumor treatments such as chemotherapy, hormonal therapy and immunotherapy may increase the occurrence of multiple primary tumors. The treatment of solid tumors, in addition to surgery, mostly adopts integrated treatment modes such as chemotherapy and radiotherapy. With the use of cytotoxic drugs such as alkylating agents or the prolongation of radiotherapy, resulting in damage to normal cells of the body and affecting DNA repair, all may increase the prevalence of hematological tumors, especially leukemia. A report by Wang Xiaojiao et al. (10) in 2019 indicated that the incidence of treatment-related leukemia in patients with breast cancer using alkylating agents was on the rise. Literature reported by Yam et al. (11) abroad in 2018 indicated that the use of alkylating agents and anthracyclines for a longer time in the treatment of malignancies may have an increased risk for AML. The combined use of alkylating agents and anthracyclines further increases the proportion of patients with solid tumors secondary to myelodysplastic syndrome (MDS) or AML if local radiotherapy is also used (12). The alkyl group of the alkylating agent is capable of forming covalent bonds with biomolecules. When the alkylating agent binds DNA, it causes strand breaks and cross-linking. Topoisomerase II inhibitors prevent DNA forming double strands, leading to the accumulation of damaged DNA and inducing the formation of free radicals that further break DNA strands. This damage may also lead to genetic changes that predispose patients to MDS and AML. The risk of multiple primary solid tumors is associated with radiotherapy (RT) and/or alkylating agent exposure, and the incidence increases over time without a plateau (13). Alkylating agents are known to have leukemogenic effects and alkylating agents or radiotherapy-associated AML are now included as a separate subtype in the World Health Organization (WHO) staging criteria for hematological neoplasms (14). MORTON et al. reported that the use of certain alkylating agents, topoisomerase II inhibitors, and platinum-based drugs, often cause fatal chemotherapy complications such as AML or MDS. Alkylating agent-associated AML is usually diagnosed 3 to 7 years after treatment of the etiology. Chromosome 5 and/or 7 abnormalities were more common in cytogenetic analysis of AML patients (15). In contrast, AML associated with topoisomerase II inhibitors has a short incubation period and is typically presented as a translocation abnormality of 11q23, 21q22, or other chromosomes (16). These mutations appear to regulate transcription of genes critical to myeloid cell differentiation, leading to abnormal fusion of chromosomes (17). It has been reported that germline mutations with BRCA 1 or BRCA 2 may be a predisposition factor for AML secondary to solid tumors, as these mutations will result in dysfunctional proteins involved in error-free repair of DNA double-strand breaks (18–20). In the SEER-Medicare database, the use of known leukemogenic drugs in initial chemotherapy, especially platinum compounds, has increased substantially since 2000, especially in gastrointestinal cancers (oesophageal, gastric, colon, and rectal cancers) (21). In this paper, platinum-based agents were used in two patients with solid tumors, one with 6 cycles of pemetrexed + cisplatin and the other with 3 cycles of oxaliplatin combined with capecitabine and 3 cycles of oxaliplatin combined with tegafur. Due to the short survival time of the two patients after APL diagnosis, no further gene mutation test was conducted to confirm whether the patients’ APL and solid tumor had the genetic susceptibility as described above. In addition to treatment-related factors such as chemotherapy and radiotherapy, patients’ own lifestyle habits such as smoking and alcohol consumption, viral infections and immune deficiencies are common factors contributing to the development of multiple primary cancers, and whether AML or APL correlates with the development of multiple primary cancers needs to be further investigated in large clinical trials.

According to the literature (22), the treatment of MPC generally adheres to the following principles: surgical resection is preferred, with every tumor removed if possible, and staged surgery if necessary; the tumors that are more malignant and more threatening to the patient are treated first, and multidisciplinary treatment is combined to optimize the treatment plan. There is no uniform standard for the treatment of MPC patients with combined APL. As APL is characterized by rapid onset, many bleedingsites and easy combination with DIC, the early use of retinoic acid is still advocated to improve the survival rate of patients when the combined multiple primary solid tumors are in a stable stage, but attention should be paid to the prevention of DIC, tumor lysis syndrome and retinoic acid syndrome. In this paper, two MPC patients with combined APL were given standard doses of retinoic acid combined with arsenic or cytarabine at the diagnosis of APL. Unfortunately, both patients developed bleeding at different sites during treatment, and their families abandoned further treatment and the two patients eventually died. Due to the low incidence and poor efficacy of AML combined with multiple primary cancers, there is no uniform treatment protocol and treatment strategies including chemotherapy, hematopoietic stem cell transplantation, immunomodulation and symptomatic support (23). Among the 12 cases of AML combined with multiple primary solid tumors reported by Mi Ruihua (6), the AML induction treatment regimen included induction remission, consolidation chemotherapy and other chemotherapy regimens commonly used in the treatment of myeloid leukemia. The overall survival [M (range)] of the 12 patients was 12.5 (3.8-48.0) months, depending on the stage of the tumor, the patient’s blood picture, coagulation function and physical condition. Because of the small sample size and the fact that all the cases were AML patients without APL, the treatment options for patients with APL combined with MPC needs to be further investigated.

The two patients with MPC in combination with APL in this paper both died eventually. The prognosis of multiple primary cancers is influenced by a number of factors, including the chronological nature of tumorigenesis (24). In a study by Ventura (25) on patients with lung cancer combined with other solid tumors, it was shown that patients with heterochronic multiple primary cancers had a higher risk of death than those with simultaneous multiple primary cancers. This may be due to the weakening of the body’s immune function as a result of receiving multiple anti-tumor treatments within a short period of time or at the same time. However, the two cases in this paper were both patients with heterochronic multiple primary solid tumors combined with APL, and the average survival time after diagnosis of APL was 14 days, which may be related to the aggressive early pathogenesis of APL disease. Therefore, the prognostic factors and survival analysis of MPC patients with combined APL still need further clinical research and analysis.

In summary, the presence of combined hematological malignancies should be considered in patients with solid tumors combined with unexplained blood changes, and prompt bone marrow aspiration and bone marrow biopsy should be performed to confirm the diagnosis. Treatment of hematological neoplasms, especially acute leukemia combined with multiple primary cancers, is challenging, and the difficulty lies in balancing different treatment modalities and risk assessment. Given the complexity of the etiology and pathogenesis of APL combined with heterochronous multiprogenitor carcinoma and the variability of clinical characteristics of patients with APL, clinical knowledge and experience is relatively limited. The treatment and survival of MPC patients with combined APL needs to be improved and enhanced.

## Data Availability Statement

The original contributions presented in the study are included in the article/supplementary material. Further inquiries can be directed to the corresponding author.

## Ethics Statement

This study was reviewed and approved by the Ethics Committee of Drug Clinical Trials of Wuwei Tumor Hospital of Gansu Province, which affiliated to Wuwei Tumor Hospital of Gansu Province, China. Written informed consent was obtained from the individual(s) for the publication of any potentially identifiable images or data included in this article.

## Author Contributions

Conception and design: CW. Acquisition of data: YS, QL, and XH. Drafting of the manuscript: YS. Critical revision of the manuscript for important intellectual content: CW. Administrative, technical, or material support: HHZ, YZ, HZ, ZY, and FG. Supervision: CW. All authors contributed to the article and approved the submitted version.

## Conflict of Interest

The authors declare that the research was conducted in the absence of any commercial or financial relationships that could be construed as a potential conflict of interest.

## Publisher’s Note

All claims expressed in this article are solely those of the authors and do not necessarily represent those of their affiliated organizations, or those of the publisher, the editors and the reviewers. Any product that may be evaluated in this article, or claim that may be made by its manufacturer, is not guaranteed or endorsed by the publisher.
